# Regulating the regulator: a survey of mechanisms from transcription to translation controlling expression of mammalian cell cycle kinase Aurora A

**DOI:** 10.1098/rsob.220134

**Published:** 2022-09-07

**Authors:** Roberta Cacioppo, Catherine Lindon

**Affiliations:** Department of Pharmacology, University of Cambridge, Cambridge CB2 1PD, UK

**Keywords:** Aurora Kinase A, oncogene, transcription, mRNA processing, translation, cell cycle

## Abstract

Aurora Kinase A (AURKA) is a positive regulator of mitosis with a strict cell cycle-dependent expression pattern. Recently, novel oncogenic roles of AURKA have been uncovered that are independent of the kinase activity and act within multiple signalling pathways, including cell proliferation, survival and cancer stem cell phenotypes. For this, cellular abundance of AURKA protein is *per se* crucial and must be tightly fine-tuned. Indeed, AURKA is found overexpressed in different cancers, typically as a result of gene amplification or enhanced transcription. It has however become clear that impaired processing, decay and translation of AURKA mRNA can also offer the basis for altered AURKA levels. Accordingly, the involvement of gene expression mechanisms controlling AURKA expression in human diseases is increasingly recognized and calls for much more research. Here, we explore and create an integrated view of the molecular processes regulating AURKA expression at the level of transcription, post-transcription and translation, intercalating discussion on how impaired regulation underlies disease. Given that targeting AURKA levels might affect more functions compared to inhibiting the kinase activity, deeper understanding of its gene expression may aid the design of alternative and therapeutically more successful ways of suppressing the AURKA oncogene.

## Graphical abstract

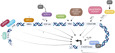

## 1. Introduction

The *AURKA* gene (also known as *STK6*, *STK15*, *IAK1*, *AIK*) encodes a member of the human Aurora family of kinases that are critical regulators of cell division. This family comprises two other members, namely AURKB and AURKC, and is characterized by a highly conserved Serine/Threonine kinase domain. First discovered using genetic screens in *Drosophila* [1], Aurora Kinase A (AURKA) phosphorylates target substrates to modulate maturation of centrosomes as well as formation of the mitotic spindle, processes that are crucial for the correct segregation of chromosomes during mitosis (M phase) [2]. Persistent association of high expression of AURKA with cancer progression, poor prognosis and drug resistance has been reported to such an extent that AURKA represents a distinguished target in the development of anti-cancer drugs [3]. In recent years, growing evidence uncovered novel cancer-promoting roles of AURKA that are kinase-independent and occur in the nucleus [4–7]. These observations fortify the concept that deregulation of expression might alone be sufficient to drive AURKA oncogenic functions, since some of these can be exerted without the need for activation of the kinase or concomitant deregulation of kinase activators. For this, suppressing its expression might represent a more efficient way to target oncogenic AURKA than using kinase inhibitors [8].

AURKA overexpression in human malignancies is mostly reported to be caused by gene amplification, enhanced transcription, or loss of miRNA-mediated silencing. However, increased AURKA protein is not accompanied by changes in mRNA abundance in some cancers [9–11]. This implies that modulation of protein stability and translation also underlie AURKA altered expression in disease, although fewer examples are available in the literature. For instance, AURKA overexpression through reduced proteolysis has been observed in head and neck [12] and breast [13] cancer cells, and a new study reports that undegraded AURKA at mitotic exit enhances fragmentation of the mitochondrial network in the following interphase [14], with fragmented mitochondrial networks being a characteristic of some cancer cells, including human invasive breast cancer [15]. Similarly, deregulation of translation also contributed to the overexpression of AURKA in some cases [16,17]. Therefore, virtually every step regulating AURKA levels has the potential to be involved in its oncogenic activation, and the control of AURKA expression is more complex than anticipated in both physiological and pathological conditions. Regardless, it is surprising how the past decade has witnessed an explosion of studies on the functions and regulation of AURKA protein, rather neglecting fundamental questions about the modulation of its gene expression. At present, if control of AURKA protein stability and degradation have been characterized to a great extent, and multiple molecular mechanisms responsible for timely transcription of *AURKA* gene are known, fewer mechanisms of AURKA mRNA post-transcriptional and translational regulation have been described. Nor has the current state of knowledge of the regulation of AURKA gene expression been comprehensively reviewed. For this reason, in this review we first elucidate the molecular mechanisms of AURKA expression at the level of DNA and mRNA, highlighting the tight link between such mechanisms and disease. Finally, we integrate the current knowledge to offer an all-inclusive view of the temporal expression of AURKA during the cell cycle.

## 2. *AURKA* gene structure

The *AURKA* gene was mapped by a fluorescence in situ hybridization (FISH) experiment that yielded a signal in chromosome 20 (20q13.2) and one in chromosome 1 (1q41) [18]. Further analyses showed that the 20q13.2 band corresponded to the functional *AURKA* gene, as later corroborated by interspecific backcross mapping [19], and that the second band represented an *AURKA* processed pseudogene on chromosome 1. Three more pseudogenes were subsequently described for *AURKA*, located on different chromosomes [20], with the one located on chromosome 1 transcribing the long non-coding RNA AURKAPS1 [21]. The *AURKA* gene is located in the reverse strand orientation and consists of a total of 11 exons and 10 introns within a region 22 948 base pairs (bp) long, spanning from location 56 369 389 bp to 56 392 337 bp (GRCh38.p13). The open reading frame (ORF) of the full-length AURKA cDNA is 1212 bp and encodes a 403-amino acid protein of approximately 46 kDa. Exons IV to VI encode the unstructured N-terminal domain, whereas exons VII to XI code for the conserved central kinase domain and the C-terminal domain. The upstream regulatory region for *AURKA* gene, which includes the promoter, extends for 4.2 kb and is shared with the *CSTF1* gene. It is worth noting that *AURKA* gene maps onto an intrinsically unstable chromosome region with frequent defects [22].

## 3. Transcriptional regulation

*AURKA* is expressed in almost all somatic cells, predominantly in dividing tissues such as haematopoietic cells, mammary gland, colon and testis. Conversely, *AURKA* expression is low in adult tissues with low or no rate of proliferation [22–24]. Low abundance of AURKA protein however does not correlate with lack of function. At least two lines of evidence support this notion, both based on discoveries of alternative non-mitotic and cell-specific functions of AURKA. Firstly, AURKA exerts important physiological functions during the interphase of cycling cells (reviewed in [25]), when its protein levels are much lower than in mitosis. Secondly, specific non-mitotic functions of AURKA have also been reported in non-cycling cells, such as neurons [26], in which the protein has intrinsically low expression levels (proteinatlas.org) [27].

**Figure 1.**
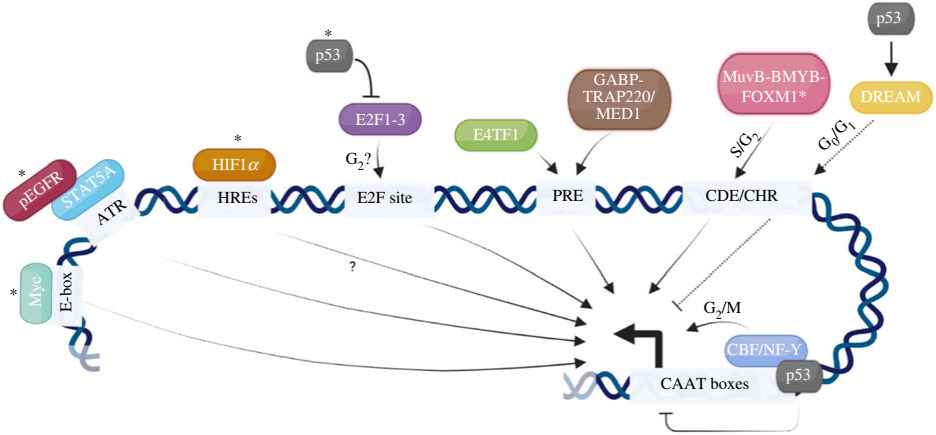
Key regulators of *AURKA* transcription. *ATR*, AT-rich region. *CDE*, cell cycle-dependent element. *CHR*, cell cycle gene homology region. *E-box*, enhancer box. *HRE*, hypoxia response element. *PRE*, positive regulatory element. G_0_, G_1_, S, G_2_, M, cell cycle phases. *, factors that also interact with AURKA protein in potential regulatory feedback loops. Thick arrow indicates start site and direction of transcription. Figure created using BioRender.com.

The existence of a variety of factors and signalling effectors that have been reported to modulate transcription of the *AURKA* gene (figure 1), both in normal and disease contexts, is very much a reflection of the plethora of cellular sources and experimental conditions adopted among the numerous studies. Several aspects of *AURKA* transcriptional regulation have been examined, and most investigations turned to classical luciferase reporters, Chromatin-immunoprecipitation (ChIP) and electrophoresis-based DNA-binding assays to uncover the minimal requirements for *AURKA* transcription, what dictates its cell cycle-dependency, and some of the transcriptional mechanisms responsible for pathological expression. However, many questions remain to be addressed (table 1). Nonetheless, one should be careful in assuming that the transcriptional mechanisms that we know today all co-exist, since many were uncovered using cancer cell lines or tissues and it is often not made clear whether the mechanisms described can be generalized to the normal context.

**Table 1.** List of questions that remain open on the regulation of AURKA gene expression in different pathological contexts, grouped by level of regulation.

level of regulation	outstanding questions	pathological context	ref.
transcription	tissue-specific effects on *AURKA* transcription in response to hypoxic conditions	hypoxia, cystic renal disease	[28–31]
	EGFR-mediated *AURKA* transcriptional regulation for cellular adaptation to EGF signalling	cancer	[32]
	mechanism of the viral early oncoprotein E6 in regulating *AURKA* transcription	HPV16-induced carcinogenesis	[33]
	EWS-Fli1-dependent transcriptional enhancement of *AURKA*	Ewing's sarcoma	[34]
post-transcription	role of exon II in regulating AURKA translation and association with tumorigenesis	breast and colorectal cancer	[11,35]
	inclusion of exon III as protective mechanism against tumorigenesis	breast cancer	[24]
	role of ERβ in controlling AS of AURKA exon II/III	breast cancer	[36]
	targeting AURKA AS via Spliceostatin A and Madrasin as a therapeutic intervention to reduce expression levels	cancer	[37–40]
	role of APA of AURKA mRNA in mediating AURKA overexpression and oncogenic activity	breast and lung cancer	[41–43]
	MCPIP1-mediated AURKA mRNA destabilization	neuroblastoma	[44]
	IGF2BP1-mediated AURKA mRNA stabilization	cancer	[45]
	miRNA-mediated targeting of AURKA mRNA	breast, liver, lung cancer	[46–49]
translation	mechanism of IRES-dependent AURKA translation	breast cancer	[16]
	combined roles of hnRNP Q1 and EGF/EGFR signalling in controlling AURKA translation	breast cancer	[17]

### 3.1. Molecular mechanisms of *AURKA* transcription

#### 3.1.1. Requirements for *AURKA* transcriptional activation

The fact that *AURKA* promoter lacks a conserved TATA-box prompted Tanaka *et al.* [20] to search for sequence elements necessary for the transcriptional activation of the human *AURKA* gene. By transiently transfecting HeLa and NIH3T3 cells with *AURKA* promoter-luciferase constructs containing a series of truncations or mutations, a positive regulatory element (PRE) (CTTCCGG, −85 to −79) was identified in the 5′ region flanking the +1 transcription start site (TSS) that is crucial for the transcriptional activity of *AURKA* gene. E4 Transcription Factor 1 (E4TF1), a member of the E26 transformation-specific (Ets) family of transcription factors, was found to bind to the PRE and to be predominantly responsible for *AURKA* transcriptional activation. In HeLa cells, deletion of the −124 to −90 sequence further decreased *AURKA* promoter activity, suggesting the presence of a *cis*-element for a tissue-specific factor that could modulate *AURKA* transcription by a yet unknown mechanism.

Another member of the Ets family of transcription factors that is thought to mediate activation of *AURKA* transcription is GA-Binding Protein (GABP), highly homologous to E4TF1 [50], in conjunction with TR-associated Protein/Mediator complex subunit 1 (TRAP220/MED1) [51]. TRAP220/MED1 is necessary for basal transcription of *AURKA*, and, again using luciferase reporter assays, it was shown that the TRAP220/MED1-mediated activation of *AURKA* transcription requires the PRE element in HeLa cells. Both TRAP220/MED1 and GABP were alone able to bind the PRE-containing region of *AURKA* promoter, and GABP binding to PRE was unaffected by TRAP220/MED1 silencing. Moreover, TRAP220/MED1 directly interacted with GABP *in vivo* and *in vitro*. From here, it was postulated that TRAP220/MED1 is recruited to *AURKA* promoter by PRE-bound GABP. These observations could be further substantiated by silencing GABP to establish that it is necessary for TRAP220/MED1 binding to *AURKA* promoter to activate transcription.

#### 3.1.2. Cell cycle periodicity of *AURKA* transcription

Although the PRE is required for *AURKA* gene transcriptional activity, it is not responsible for its cell cycle-dependent regulation. For example, in electrophoretic mobility shift assays (EMSAs), E4TF1 remained bound to *AURKA* PRE throughout the cell cycle [20]. On the other hand, GABP has been reported to regulate genes in a cell cycle-dependent manner [52], but whether this is also the case for *AURKA*, and whether it also relies on the PRE, is not clear. Most genes whose expression peaks in G_2_ (late genes), such as *Cyclin A*, *cdc2*, *cdc25C* and *Plk*, are transcriptionally repressed in G_0_ and early G_1_, with their promoters being relieved from repression in late G_1_/early S phase [53,54]. In addition to de-repression, promoters of ‘late genes’ also undergo activation late in S phase, which is sustained until M phase [55]. A dual sequence module that consists of a Cell Cycle-Dependent Element (CDE) and a Cell Cycle Gene Homology Region (CHR) is crucial for this regulation: the CDE/CHR module is responsible both for transcriptional repression in G_0_ and early G_1_ and for transcriptional activation later in S phase, depending on the protein composition of CDE/CHR-binding Multi-vulval class B (MuvB)-based complex (figure 1). Additionally, transcriptional activation of G_2_/M genes is also mediated by CCAAT-boxes, recognized by the CCAAT-Binding Factor/Nuclear transcription Factor Y (CBF)/(NF-Y) complex.

##### 3.1.2.1. *AURKA* transcriptional repression in G_0_ and early G_1_

Because of its transcriptional time window in dividing cells, *AURKA* is historically classified as a ‘G_2_/M’ gene [56,57] and, just downstream of the *AURKA* PRE, a CDE (−44 to −40) and a CHR (−39 to −35) are located. However, dissimilar to canonical CDE/CHR sites, *AURKA* CHR sequence (5′CTTAA3′) diverges from the consensus (5′TTTGAA3′) and CDE and CHR are located next to each other without the typical 4 nucleotides (nt) spacer. Mutations in the CDE or CHR on *AURKA* promoter-containing reporter constructs increased G_1_-specific transcription, suggesting that the CDE/CHR module functions as repressor of *AURKA* transcription in G_1_ [20]. As mentioned above, the transcriptional outcome dictated by CDE/CHR site depends on the contextual partners of the CDE/CHR-binding MuvB sub-complex. In G_0_ and early G_1_, *AURKA* CDE/CHR is expected to be bound by a complex comprising the core MuvB sub-complex (LIN9, LIN37, LIN52, LIN54, RBBP4) plus p130 or p107, DP, E2F4 and E2F5 factors (altogether the DREAM complex) [58–60]. In particular, the DREAM complex binds to the CDE sequence through E2F4 and E2F5, and to the CHR sequence through MuvB, resulting in transcriptional repression [61,62], although the precise mechanism through which this occurs is still enigmatic.

##### 3.1.2.2. *AURKA* transcriptional activation in S to M

Once cells are in S phase, *AURKA* transcriptional program switches from repression to activation, and positive transcriptional activity lasts until M phase. It has been estimated that in NIH3T3 cells *AURKA* is transcribed at a rate of 5.39 mRNA molecules/(cell × hour) [63]. Contributing to *AURKA* activation are three classes of sequence motifs: CDE/CHR module, E2F sites and CCAAT-boxes. As we will explore, *AURKA* activation through CDE/CHR and CCAAT motifs has been delineated, whereas the role of E2F sites, as well as the functional interaction between the different classes of motifs, is still debated.

The suppressor DREAM complex is thought to disassemble from the CDE/CHR in G_1_ [61]. However, the activator complex initiates assembly onto the CDE/CHR site only in S phase [59,64,65], leaving a time window between release of DREAM complex and activation of transcription. This de-repression that precedes activation might be responsible for initial low-level transcription. Accordingly, AURKA mRNA molecules can be minimally detected already in G_1_ phase [24]. The *AURKA* CDE/CHR-binding activator complex comprises the MuvB sub-complex this time associated with other partners: B-MYB and Forkhead Box M1 (FOXM1) [62]. It is a matter of debate whether B-MYB and FOXM1 directly bind DNA when they are in complex with MuvB, and to which sites [61]. In S, MuvB first recruits B-MYB and this allows subsequent recruitment of FOXM1 in early G_2_ (MuvB-BMYB-FOXM1 complex). During the increase of *AURKA* transcription at the S/G_2_ transition, B-MYB undergoes phosphorylation and consequent proteasomal degradation, enabling the hyper-phosphorylation and full activation of FOXM1 in G_2_. In this way, transcription is only maximally activated by MuvB-phosphoFOXM1 complex after B-MYB is degraded, and the former is thought to sustain *AURKA* transcription until M phase [61,64,65]. However, analysis of global nuclear run-on followed by RNA sequencing (GRO-seq) on RNA extracted from thymidine- and nocodazole-blocked cells (synchronized in S and M phases, respectively) suggests that transcription of G_2_/M genes may be maximal already in S phase, and it is the steady-state mRNA levels that peak in M [66] (see §6).

E2F transcription factors include both activators (E2F1, E2F2 and E2F3) and repressors (E2F4 and E2F5) that recognize specific E2F sites (consensus 5′TTTCCCGC3′), although E2F4 and E2F5 can also bind CDEs (see above). E2Fs 1-3 typically concur to activate transcription of G_1_/S genes but have also been found implicated in the activation of G_2_/M genes in late S or G_2_ [67–69]. Upstream of PRE, two sites on *AURKA* promoter (−307 to −302 and −260 to −254) resemble the conserved binding site for E2Fs. This fostered the hypothesis that *AURKA* could be directly induced by E2Fs. However, *AURKA* gene was neither found among E2F1- or E2F2-induced genes nor among E2F3-induced genes in two studies that used similar DNA microarray methods to profile transcriptome changes following E2F1, E2F2 or E2F3 overexpression [67,68]. Nevertheless, this could be due to the stringency of criteria set for identifying E2F targets, such as expression fold change, if only minor transcriptional activation of *AURKA* was induced*.* In accordance with this supposition, *AURKA* promoter was later found to be bound weakly by E2F1, E2F2 and E2F3 in ChIP assays, albeit this result could be biased by lack of cell synchronization [70]. Suitably, E2F3 showed no binding to the same *AURKA* promoter region when this was assessed performing a ChIP experiment from cells synchronized in S [71]. One explanation for these disparate results could be that E2F3 binds to *AURKA* to activate transcription only in G_2_ but only to a limited extent, as observed for other G_2_/M genes [69]. Nevertheless, in other reports *AURKA* transcription has been shown to be induced by E2F1 following low concentration of arsenic treatment in immortalized keratinocytes and bladder cells [72,73]. In sum, these observations point to the conclusion that *AURKA* is likely to be positively but modestly regulated by E2F1-3, possibly through the two putative E2F sites, and this regulation probably adds to the MuvB-BMYM-FOXM1-mediated transcriptional activation.

The third known mechanism of *AURKA* cell cycle-dependent transcriptional activation relies on the two CCAAT-boxes on its promoter (−4 to +1 and +29 to +33), separated by a conserved spacer of approximately 30 bp. Using a dominant-negative approach, in conjunction with DNA-binding and luciferase assays, Hu *et al.* [71] demonstrated that the CBF/NF-Y transcription factor complex is needed for G_2_/M progression. It induces expression of late genes, including *AURKA*, and both CCAAT-boxes on *AURKA* promoter are needed for this induction. Even so, CBF/NF-Y was bound to these sequence elements throughout the cell cycle, and the authors could not explain the molecular mechanism that conferred temporal specificity to the CBF/NF-Y-mediated transcriptional activation of *AURKA*. It has been reported that CBF/NF-Y binding to rat *CDK1* CCAAT-box is necessary for recruitment of activator E2Fs to their respective sites on *CDK1* promoter in S phase to induce transcription [69]. A similar mechanism could apply to *AURKA*, although it is yet to be investigated. It is likely that CBF/NF-Y mediates *AURKA* activation by directly recruiting the RNA-Polymerase II [74] and/or by recruiting enzymes for positive chromatin modifications (see below). However, such processes need further study to better frame CBF/NF-Y-mediated *AURKA* activation.

Once AURKA mRNA levels reach a peak in M, transcription is switched off. To this end, FOXM1 is ubiquitinated through FZR1-activated Anaphase-Promoting Complex/Cyclosome (APC/C^FZR1^), which leads to its proteasomal degradation [75]. This contributes to decreasing AURKA mRNA levels at mitotic exit [61]. However, only in G_1_ does repression of transcription actively take over through the mechanisms discussed.

### 3.2. Signalling pathways modulating *AURKA* transcription

In addition to intrinsic cell cycle-dependent regulation, *AURKA* transcription is also modulated in response to internal and external stimuli, such as DNA damage, growth factors and environmental cues, both in normal and pathological conditions, ultimately offering a means of cell cycle control.

An important regulator of *AURKA* transcription is the p53 tumour suppressor, which represses *AURKA* expression following DNA damage through multiple mechanisms. Firstly, p53 is able to activate the DREAM complex to block transcription of G_2_/M genes outside G_1_ in the event of DNA damage [62]. Secondly, via the p53-Rb-E2F3 axis, p53 activation upon DNA damage increases the level of p21, reducing the activity of Cyclin-Dependent Kinase 2 (CDK2) and therefore blocking Retinoblastoma protein (Rb) hyper-phosphorylation, in turn promoting the sequestering of transcription factor E2F3; E2F3 becomes unable to bind to *AURKA* promoter at the CDE/CHR site, and this prevents activation of *AURKA* gene transcription [70]. Thirdly, p53 has been shown to constitutively interact with CBF/NF-Y at CCAAT-boxes of G_2_/M genes, and, upon DNA damage, it is rapidly acetylated resulting in release of Histone Acetyl-Transferases (HATs) and recruitment of histone deacetylases (HDACs) on the promoters, which induces transcriptional repression [76].

The critical transcription factor Myc has also been found implicated in positively regulating *AURKA* transcription. This is directly mediated by Myc binding to Enhancer-boxes (E-boxes) on *AURKA* promoter in mouse [77] and human [78] cells. Interestingly, Myc and its binding partner Max are associated with the *AURKA* promoter during G_2_. It seems that such association is prevented by topoisomerase I inhibition and results in downregulation of AURKA expression. With AURKA being implicated in centrosome dynamics, the study reported that topoisomerase I inhibition prevented separation of centrosomes, leading to G_2_ arrest and cellular senescence. Therefore, a model was proposed in which Myc bridges *AURKA* transcription to mechanisms of sensing DNA status [78].

A link between the hypoxia response and *AURKA* expression was first uncovered by a group that observed increased AURKA mRNA levels upon hypoxia in HepG2 hepatoma cells, and this occurred via Hypoxia-Inducible Factor 1 (HIF-1*α*) [28]. The *AURKA* promoter contains three hypoxia response elements (HREs) at positions −336 to −332 (HRE-1), −323 to −319 (HRE-2) and −240 to −236 (HRE-3), but HIF-1α-dependent induction of a luciferase reporter was most sensitive to mutation of HRE-2, suggesting that HRE-2 functionally represents the major HIF-1*α* binding site. In addition, *AURKA* silencing inhibited hypoxia-induced proliferation of HepG2 cells, suggesting that *AURKA* transcriptional up-regulation in hypoxic conditions is involved in controlling HepG2 cell proliferation. These results were later validated by Cui *et al.* [29], who also showed that HIF-1*α* induced *AURKA* expression by recruiting HATs to its promoter. In addition, they observed that expression of HIF-1*α* positively correlated with AURKA expression in hepato-cellular carcinoma (HCC) tissues. However, in other studies using cancer cells, hypoxia contrarily induced a down-regulation of *AURKA* expression [30], suggesting that the outcome on *AURKA* transcription in response to hypoxia might be tissue-specific. *AURKA* transcription is also up-regulated in cystic renal diseases where function of the HIF-1*α* destabilizer factor von Hippel-Lindau (VHL) is lost, and where formation of primary cilia and motility of renal cells is altered [31]. The regulation of *AURKA* expression by HIF-1*α* might occur not only in conditions of hypoxia, but also under other conditions that activate HIF-1*α* regardless of oxygen levels in the tissue environment, for example, under the influence of hormones and growth factors, cytokines, or other stresses. In summary, HIF-1*α* is a relevant factor linking *AURKA* expression to environmental cues.

A connection between *AURKA* transcription and growth factor signalling has also been reported [32]. In transformed cells with overexpression of epidermal growth factor (EGF), transcription of *AURKA* was enhanced following translocation of EGF receptor (EGFR) into the nucleus, where it is activated by phosphorylation. Phospho-EGFR then binds to *AURKA* promoter and facilitates its transcription. Since EGFR lacks a DNA-binding domain, its binding to *AURKA* promoter occurs via Signal Transducer and Activator of Transcription 5A (STAT5A), which is recruited to the AT-rich (ATR) region in the upstream sequence of *AURKA*. However, although some of these findings were replicated in multiple immortalized and cancer cell lines, it is not clear how cells use this EGFR-mediated *AURKA* transcriptional regulation to adapt their proliferation rates to EGF signalling.

*AURKA* is also a target for the oncogenic Human Papillomavirus 16 (HPV16) in cell carcinogenesis, due to the involvement of the viral early oncoprotein E6 in elevating *AURKA* transcription [33]. Furthermore, *AURKA* was found transcriptionally enhanced by Ewing sarcoma breakpoint region 1-Friend Leukaemia Integration 1 (EWS-Fli1) fusion protein, which results from a chromosomal translocation, in Ewing sarcoma cells following EWS-Fli1 binding to a Ets-binding site at −84 to −71 [34]. It would be interesting to test whether abnormal cellular phenotypes caused by EWS-Fli1 could be rescued by *AURKA* silencing.

### 3.3. Epigenetic regulation

In addition to the different transcription factors that regulate *AURKA* gene expression, greater fine-tuning at the transcriptional level is brought about by post-translational modifications of histones residing in proximity of the *AURKA* promoter. It is now well known that chromatin modifications affect expression of virtually all eukaryotic genes [79]. The observation that HDAC inhibitors diminished *AURKA* expression in lung cancer cells supports the idea that *AURKA* transcription is regulated by epigenetic mechanisms [80]. Indeed, it is known that activation of G_2_/M genes is linked to acetylation of promoter histones and nucleosome positioning, mediated by enzymes recruited by the diverse transcription factor complexes that bind promoter elements, including CBF/NF-Y and MuvB-BMYM-FOXM1 complexes [61]. It has been suggested that CBF/NF-Y binding to CCAAT-boxes allows recruitment of the p300 HAT and formation of an open chromatin state on target promoters [81,82]. However, it seems that, in this process, a distance between CCAAT-boxes of 33 bp is required to enable the correct orientation of the respective binding factors, whereas that between CCAAT-boxes on *AURKA* promoter is shorter. Other evidence links the MuvB-BMYM-FOXM1 complex to chromatin modifications, for example, deletion of BMYB resulted in reduced histone acetylation on *AURKA* promoter in cells entering the cell cycle [64].

Direct analysis of the relationship between epigenetic modifications on *AURKA* promoter and transcriptional activity has been investigated in many cancer contexts. AT-Rich Interactive Domain 1A (ARID1A), a component of the Switch/Sucrose Non-Fermentable (SWI/SNF) chromatin-remodelling complex, which assembles nucleosomes to discourage access of transcription factors to chromatin, has been found to occupy the *AURKA* promoter and negatively regulate its transcription in colorectal cancer cells [83]. Others observed that p53 directly binds to an upstream region of the *AURKA* promoter *in vivo* and represses transcription through the recruitment of HDAC1 and of the mSin3 corepressor in non-small-cell lung cancer [84]. Furthermore, it was shown that INI1/SNF5, core component of the mammalian SWI/SNF complex, repressed *AURKA* transcription in rhabdoid but not in non-rhabdoid tumour cells, as it associated with *AURKA* promoter only in the former case [85]. These might only be few of the examples of how chromatin modifications and modifiers control *AURKA* expression in disease.

In conclusion, the number and variety of mechanisms discussed reflect how important it is for the cell to exercise a tight control of *AURKA* transcription. Despite this, characterization of *AURKA* transcription lags behind that of other cell cycle regulators such as Cyclins. *AURKA* is regulated by transcription factors belonging to the Ets family, such as EGFR, GABP, E4TF1 and many more unidentified factors. Most of these factors use *AURKA* promoter elements such as PRE, HRE, CDE and CHR, lying within a 400 bp region upstream of the coding sequence. However, only a fraction of the total 4.2 kb region immediately upstream *AURKA* TSS has been analysed, thus the presence of other regulatory elements further upstream is not to be excluded. In addition, the integrated function of all the different sequence elements is not known and constitutes a fundamental research quest of a complete framework of *AURKA* transcriptional regulation. It is also important to note the recurring phenomenon in which transcription of *AURKA* is regulated by some factors that AURKA engages with in protein-protein interactions, such as p53 [86], HIF1 [87], EGFR [88], Myc and FOXM1 [7] (figure 1). This suggests the presence of uncharted regulatory feedback loops, the exploration of which may reveal integrative circuits that control critical cellular functions, in addition to being exploited for therapeutic purposes [89,90].

## 4. Post-transcriptional regulation

The processing of *AURKA* gene transcript results in a precursor-mRNA (pre-mRNA) of length 2–2.4 kb. The events of AURKA pre-mRNA splicing and polyadenylation, which are, respectively, addressed in this section, are subject to regulation, leading to a heterogeneity of alternative mRNA isoforms that differ for the length and content of both untranslated regions (UTRs). Although genome-wide studies hint that splicing and polyadenylation are relevant steps of AURKA mRNA regulation, we still do not know how they affect AURKA expression and/or function in detail. Our final, short section on mechanisms controlling AURKA mRNA stability reflects how this aspect of AURKA mRNA regulation has been little explored. In future, the development of reporter assays that bypass transcriptional regulation to selectively focus on post-transcriptional events might be a key strategy to investigate AURKA mRNA modulation *in vivo*. Such assays could also offer a platform to screen for regulators or modulator drugs [91,92]. In addition, the large collection of RNA-sequencing data from different tumour types alongside clinical profiles of patients contained in The Cancer Genome Atlas (TCGA) could provide ample starting material to systematically study the biological significance of AURKA mRNA isoforms and their association with disease.

### 4.1. Pre-mRNA splicing

Early studies characterizing *AURKA* expression hinted that more than one transcript exists for *AURKA* [22,93], although, at the time, low-resolution Northern and RT-qPCR methods, mixed with relatively less standardized experimental conditions (i.e. RNA extraction methods, instruments, probe design, etc.), prohibited systematic studies of isoform expression among different cell types and tissues. Most of our knowledge on AURKA splicing derives from larger studies of high-throughput RNA sequencing or splicing-sensitive genome-wide microarrays. However, these are not conducive to a unifying hypothesis for the splicing regulation of AURKA, both physiological and disease-related, hence our sometime fragmented discussion on the topic.

**Figure 2.**
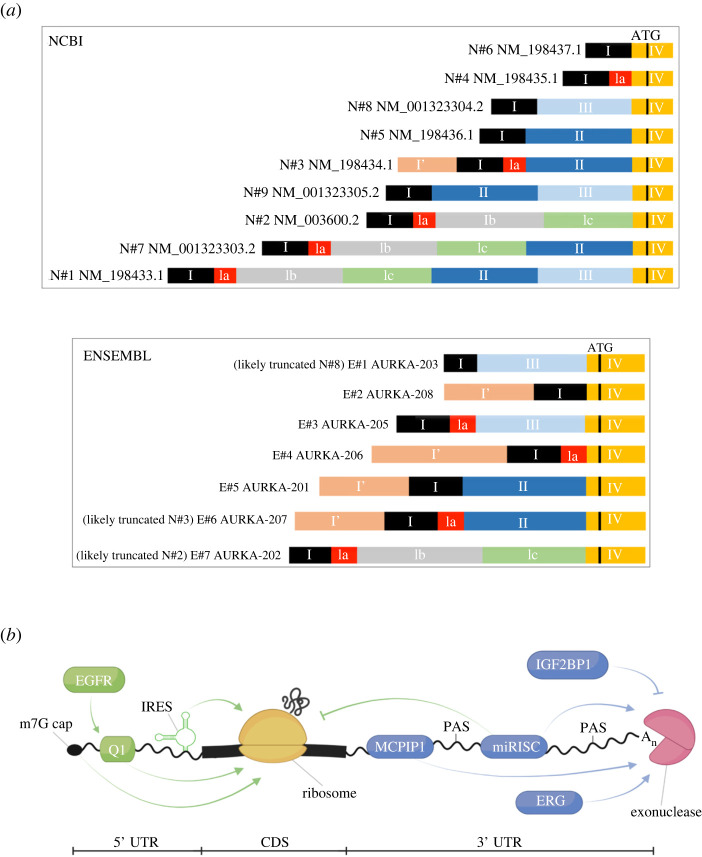
Post-transcriptional regulation of AURKA mRNA. (*a*) Annotated 5′UTR splicing isoforms. (*b*) Regulators of AURKA mRNA translation (green) and decay (blue). *I–IV*, exons. A_n_, polyA tail. *ATG*, start codon. *CDS*, coding sequence. *m7G*, 7-methyl-guanosine. *miRISC*, miRNA-induced silencing complex. *PAS*, polyadenylation site. *UTR*, untranslated region. Figure created using BioRender.com.

Sixteen different high-scoring transcript isoforms resulting from alternative splicing (AS) have been annotated so far, 9 of which are listed in the NCBI database (N#1–9) and 7 in the Ensembl database (E#1–7) (figure 2*a*). All 16 isoforms are 5′UTR splicing variants, mainly via alternative splice sites and exon skipping, whereas the coding sequence and the 3′UTR follow canonical splicing. The fact that no Matched Annotation between NCBI and EBI (MANE) label was given to any of the 16 isoforms indicates that they are distinct transcripts independently annotated by Ensembl and NCBI. Other truncated and/or incomplete transcripts for *AURKA* gene are annotated, but since they are given a low annotation score and are not fully validated, they were not included in our discussion.

Given that the splicing process is tightly coupled to transcription, and given the cell cycle periodicity of *AURKA* transcription, it is not surprising that *AURKA* is among genes undergoing periodic AS during the cell cycle, presumably with retention of exon III occurring as early as G_2_ [94]. This inclusion event is likely to be important for engagement of ribosomes and translation [95], and is in accordance both with reports of a general coupling between AS and translation [96,97] and with some observations that AS is inhibited during M phase [98]. Nevertheless, AURKA AS was found to be regulated neither by CDC Like Kinase 1 (CLK1) nor by SON, a large Ser/Arg-rich protein, which are two known regulators of AS of genes with crucial roles in cell cycle control [94,99,100]. What is so peculiar about AURKA transcripts or transcription that sets the regulation of its AS apart from that of other cell cycle genes? Detailed mapping of splicing sites and splicing-associated sequence elements, together with addressing the mechanistic interaction of splicing factors with AURKA transcripts, could provide some clear answers.

Different studies seem to conclude that AURKA AS plays a role in cancer, although none go so far as reporting an AURKA AS-dependent mechanism of pathogenesis [101]. Shin *et al*. [35], who were the first to report a link between AURKA AS and disease, detected three 5′UTR splicing isoforms (N#1, N#2, N#7) (figure 2*a*) in a breast cancer cell line, whereas a normal cell line only expressed N#2. Because this is the only one of the three transcripts to lack exon II, the authors inferred that this exon might be implicated in tumorigenesis. However, all three AS isoforms supported equal AURKA protein translation, apparently excluding that exon II could account for AURKA protein overexpression in breast cancers. It is worth noting though that the *in vitro* translation assay used—in rabbit reticulocyte lysate—may not recapitulate the translational regulation of AURKA mRNA occurring in breast cancer cells. A separate study found that whereas overexpression of Serine(S)/Arginine(R)-rich Splicing Factor 1 (SRSF1), known to couple AS to translational regulation, correlated with inclusion of exon II on AURKA mRNA, AURKA was not amongst translational targets of SRSF1, adding evidence to the lack of correlation between exon II and translational regulation [97]. It can therefore be presumed that the two mechanisms of SRSF1-mediated AS and translational control are uncoupled in the case of AURKA mRNA. Contrary to these conclusions however, an exon II-dependent mechanism of AURKA translational activation was proposed for colorectal cancers in which both EGFR and AURKA are overexpressed [11]. In this work, two exon II-containing transcripts (N#3 and N#5) (figure 2*a*) were the major AURKA splicing isoforms expressed in human colorectal cancers. This study interestingly shows that exon II enables AURKA mRNA to become responsive to EGF stimulus, resulting in AURKA translational up-regulation. The result could perhaps explain why exon II-dependent AURKA overexpression could not be detected using *in vitro* translation assays.

Other AURKA exons have also been found to correlate with disease. Li *et al*. [24] used splicing-sensitive microarrays to analyse AS events of genes reported to play a role in cancer. They suggest that MDA-MB-231 breast cancer cells tend to splice AURKA pre-mRNA in a way to skip exon III, arguing that exon III might provide a protective function against tumorigenesis. MCF7 breast cancer cells also skipped exon III, although to a lesser extent. When both cell lines were grown in 3D culture, they now observed that exon III was more prevalently skipped in MCF7 cells compared to MDA-MB-231 cells, indicating that culture conditions and cellular environment could regulate AURKA splicing process. However, it would be interesting to assess whether the skipping of exon III in breast cancer cell lines is linked to the splicing dynamics of exon III occurring at G_2_/M [94] (see above) and thus linked to the cell cycle.

Dago *et al.* [36] report that stable expression of Estrogen Receptor β (Erβ) in MCF7 breast cancer cells induced skipping of exon II or III in different AURKA mRNA isoforms after oestradiol treatment. A truncated form of ERβ containing only the C-terminal domain could mediate skipping of exon III in transcript N#1 only, whereas a truncated form of ERβ containing only the N-terminal domain mediated the skipping of exon III in E#205, as well as skipping of exon II in isoforms N#3 and E#201 (figure 2*a*). This might suggest that ERβ C-terminal and N-terminal domains interact with the splicing machinery in different manners. Furthermore, the study reckoned that *AURKA* gene contains ERβ binding site(s), but surprisingly *AURKA* transcription was not itself regulated by ERβ. It is therefore unknown how ERβ mechanistically controls AURKA AS.

Other studies of AURKA AS shed light onto the possibility of targeting AURKA AS as a therapeutic intervention to control its expression levels. For example, it is known that skipping of exons VI to VIII leads to formation of a premature Stop codon that consequently triggers the Non-sense Mediated Decay (NMD) pathway of AURKA mRNA degradation: a mechanism that cells potentially put in place to prevent aberrant expression of AURKA proteins [37]. Insightfully, the same study also observed that skipping of exons VI–VIII can be induced by Spliceostatin A (SSA), through inhibition of the splice-site recognition Spliceosome Factor 3B 1 (SF3B1). This resulted in lower AURKA expression and constitutes an example of drugs that aim to reduce protein overexpression by means of modulating mRNA splicing to induce mRNA decay. Consistent with this finding in HeLa cells, other studies in K562 myelogenous leukaemia cells also observed skipping of exons VI to VIII on AURKA mRNA following SF3B1 silencing and mutation [38,39]. However, it remains to be seen whether SSA-mediated AURKA silencing can function in suppressing aberrant cell behaviour. Madrasin is a second drug shown to promote exon skipping (exon X) on AURKA mRNA in different cell lines [40]. It is possible that the induced defective splicing interferes with AURKA expression, but this remains to be investigated. It is however worth noting that madrasin induced cell cycle arrest at a lower concentration than that needed to induce exon X skipping. In other reports, AURKA was recovered among the top 50 transcripts encoding proteins regulating cell growth and survival that lacked exons as a result of exon skipping events, following treatment with CLK inhibitors [102]. Also in this case, it is conceivable that the frameshift caused by exon skipping may introduce premature Stop codons and thus enable faster degradation rate of the transcript.

To summarize, inclusions of exon II and of exon III seem to be linked to cancer, for example as they render AURKA mRNA responsive to growth factors signalling. It is however not to exclude that these two exons also play a role in normal AURKA expression, as may be the case for retention of exon III at G_2_/M. Furthermore, specific splicing events of AURKA mRNA, like skipping of exons VI to VIII, could be exploited therapeutically to promote mRNA decay and control AURKA expression levels. Curiously, recent new evidence has uncovered a role for AURKA in regulation of splicing. AURKA phosphorylates core proteins of the spliceosome *in vitro* and interacts with factors that regulate the spliceosome. In addition, AURKA promotes *in vitro* splicing of the β-globin pre-mRNA and inhibition of AURKA changed the AS of different genes [103]. It was not investigated if AURKA itself is among mRNAs whose splicing is altered by AURKA protein inhibition, which may represent the first evidence for an AURKA autoregulatory mechanism acting upon its mRNA.

### 4.2. Pre-mRNA cleavage and polyadenylation

The pre-mRNA processing step of cleavage and polyadenylation is mediated by Poly-Adenylation Signal (PAS) sites located within the 3′UTR. Two canonical PASs (5′AAUAAA3′) can be found in the AURKA 3′UTR (polyasite.unibas.ch [104]) (figure 2*b*). As a consequence of tandem 3′UTR Alternative cleavage and PolyAdenylation (APA), two mRNA isoforms that differ in 3′UTR length exist for AURKA mRNA. It has yet to be investigated which AURKA PAS site is preferentially used in which cellular context and to what extent, or whether a 3′UTR isoform switch is modulable. This information might be available from systematic searching for AURKA APA events within the many APA databases available in the literature, including deep learning predictive models [105,106] or libraries created from very diverse biological and pathological contexts such as cellular stress [107,108], immune cells [109,110], cellular senescence [111], cancer [112–114], embryonic development [115] or others [116–118]. More recently, AURKA was classified within the TNBC APA subtype with the highest median index of 3′UTR shortening events, and this correlated with increased AURKA gene expression [41,119]. This TNBC APA subtype also showed a high-intensity nuclear Ki-67 staining indicative of highly proliferative nature, and patients in this subtype had the worst disease-free survival [41]. Moreover, AURKA overexpression in TNBC identifies as a factor of early recurrence and poor prognosis [42] and AURKA showed 3′UTR shortening in poor-prognosis patients of both breast and lung cancer [43]. In their quest to define a periodically regulated AS program linked to the cell cycle, Dominguez *et al*. [94] preliminarily uncovered 94 genes involved in the cell cycle and/or proliferation that undergo periodic APA during the cell cycle. Although *AURKA* is not displayed in their gene list, potentially suggesting that AURKA APA is not temporally controlled, a more in-depth analysis using updated software tools and annotations may be required. Nonetheless, the existence of two PAS sites on AURKA mRNA suggests that APA could serve to regulate AURKA expression.

Following cleavage at PAS sites, the AURKA mRNA is polyadenylated at the 3′end. The poly(A) tail enables stability and translation of mRNAs [120]. Park *et al*. propose that reduction in poly(A) tail length is coupled to translational suppression at mitotic entry only for poly(A) tails under approximately 20 nt [121]. From TAIL-seq analysis of the somatic cell cycle, they also revealed that AURKA mRNA has a median poly(A) length of 65 nt in S phase and of 59 nt in M phase, well above the approximately 20 nt median length threshold for the above correlation to occur. Such poly(A) tail length may however still be responsible for basal stability and translation of AURKA mRNA [122]. In sum, it is currently not known whether the poly(A) tail dynamics of AURKA mRNA is functional in regulating AURKA expression.

### 4.3. Regulation of mRNA stability and decay

Changes in abundance of AURKA mRNA are not exclusively due to activation or suppression of transcription. Events of mRNA stability control and decay also need to be accounted for when measuring overall mRNA levels (figure 2*b*). Like most mRNAs, that of AURKA might contain several sequence elements and structural motifs in the UTRs that dictate mRNA stability. However, to our knowledge no functional analysis providing a comprehensive view of AURKA UTR regulatory elements has been carried out to date.

AURKA mRNA average copy number has been estimated at 24 molecules/cell, and the transcript half-life around 5 h, in a population of non-synchronized cells [44,63]. Some evidence shows that depletion of the transcription factor ERG reduced decay of AURKA and AURKB mRNAs in S phase and caused accumulation of both transcripts in G_2_ and at mitotic entry, resulting in premature and higher induction and activation of AURKA and AURKB proteins, and consequential mitotic defects [123]. Collectively, the study clearly establishes that ERG-mediated degradation of AURKA and AURKB mRNAs is important to ensure proper cell cycle progression, although it does not discriminate between the individual involvement of AURKA and AURKB mRNA degradation. A recent study suggested that overexpression of the ribonuclease Monocyte Chemotactic Protein-1-Induced Protein-1 (MCPIP1) leads to the destabilization of AURKA mRNA in neuroblastoma cells, as it binds to and cleaves AURKA 3′UTR, although the precise mRNA sequence responsible for the observed interaction is still undetermined [44]. Moreover, enhanced CLIP (eCLIP) was used to validate that AURKA mRNA is a direct target of IGF2 mRNA-Binding Protein 1 (IGF2BP1), an important tumour and stem cell fate regulator, and is stabilized by IGF2BP1 binding [45]. IGF2BP1 could therefore promote AURKA oncogenic gene expression in a 3′UTR-dependent manner.

Evidence of microRNA (miRNA)-mediated regulation of the cell cycle continues to grow [124]. At least 50 different miRNAs are predicted to target AURKA 3′UTR, according to the microRNA target prediction database miRDB (mirdb.org), although only few have been validated as direct regulators of AURKA mRNA. Interestingly, many cases of miRNA targeting seem to be relevant particularly in those cancers for which AURKA overexpression is considered a promoting factor and a marker of poor prognosis, such as breast cancer, HCC and lung cancer. For example, Fadaka *et al*. [46] use molecular docking to suggest that AURKA might be regulated by miR-32-3p in breast cancer, and analyse binding energy and specific miRNA–mRNA interactions, although they did not directly probe the targeting. In HCC, miR-490-3p and miR-26a-5p could silence the expression of AURKA, allowing suppression of proliferation and migration properties of HCC cells [47], as well as reduction of chemoresistance [48]. Furthermore, it was demonstrated that up-regulating the expression of miR-32 via administration of tanshinone, could suppress AURKA expression leading to inhibition of Non-Small Cell Lung Cancer (NSCLC) [49]. These are only a few examples of direct miRNA targeting of AURKA mRNA, but many more are clearly coming, since the validation of miRNA targets can be achieved using simple and reliable methods such as *in vivo* 3′UTR reporter assays. Some interesting questions, such as the link between AURKA APA and miRNA targeting, remain—for now—totally unexplored. Since APA allows for sequences on the 3′UTR to be contextually displayed or removed, it potentially influences the targeting ability of miRNAs, offering a further layer of AURKA gene expression regulation [125].

RNA modifications also seem to have a role in influencing mRNA stability. For example, *N*^6^-methyladenosine (m^6^A) is selectively recognized by the human YTH Domain Family 2 (YTHDF2) ‘reader’ to positively regulate mRNA degradation [126]. AURKA mRNA is subject to m^6^A within the 3′UTR close to the STOP codon and is a high confident YTHDF2 target [127,128], although no significant m^6^A enrichment was detected in AURKA mRNA in any of the cell cycle phases. Nor did the mRNA display significantly higher accumulation in the absence of YTHDF2, suggesting that AURKA mRNA might be recognized by YTHDF2, but this does not mediate its degradation at any time during the cell cycle [128]. Therefore, the role of m^6^A in AURKA mRNA regulation is still unknown.

Our discussion on AURKA mRNA regulation makes evident that the study of this area has the potential to uncover novel exciting mechanisms of AURKA expression-dependent pathogenesis (table 1). It is easy to imagine that AS and APA processes are combined, and this would give rise not only to an even higher total number of isoforms for AURKA mRNA, but also to additional regulatory mechanisms of mRNA stability and translation. However, much more research is needed to fully characterize the repertoires of AURKA transcript isoforms in both physiological and pathological contexts. It has also been shown that proteins can acquire different localizations and functions depending on which 3′UTR isoform they are translated from [129–132]. For this reason, it is worth investigating whether changes in 3′UTR resulting from APA also affect AURKA protein stability, localization or function. Such a quest would show for the first time if and how 3′UTR APA controls AURKA properties.

## 5. Translational regulation

Regulation of *AURKA* gene expression at the level of translation has been less widely reported and little is known compared with its transcriptional and post-transcriptional mRNA processing. Nonetheless, it is now established that dysregulation of translation can also be linked to disease and the contributions of aberrant translation to cancer phenotypes are increasingly recognized [133–135]. The number of cell cycle regulators reported to be abnormally upregulated at the translational level in disease also includes AURKA, as we will argue below. Most of our knowledge on the topic derives from studies that make use of various genome-wide methods, which in recent years have generally shed light on translational control during the cell cycle [136–138]. On the other hand, only few are the studies conducting gene-specific investigations on AURKA translational regulation. In this section, we will focus our discussion initially on the temporal translation of AURKA in relation to the cell cycle, to then consider mechanisms known to modulate AURKA translation that are also linked to disease (table 1).

### 5.1. Cell cycle periodicity of *AURKA* translation

The average translation rate for AURKA mRNA was calculated to be 14 proteins per mRNA per hour [63]. However, such analysis, consisting of simultaneous measurements of absolute mRNA and protein copy numbers, as well as turnover rate of both, was carried out in exponentially growing mouse fibroblasts, averaging out any changes of AURKA translation rate through the cell cycle. Because AURKA protein levels peak at late G_2_ until M, when AURKA then starts to disappear, this would reasonably represent the period of highest translational activity of the AURKA mRNA before it shuts down in M phase. Accordingly, it has been known for many years that the global rate of protein synthesis is markedly reduced (by approx. 75%) in M phase compared to that in interphase [139–143]. However, this notion has become an issue of debate in recent years, following other studies that report smaller or minor variations in global translation rates between M phase and the rest of the cell cycle, possibly due to different cell synchronization methods [144–149]. Nonetheless, translation of hundreds of mRNAs is found to be specifically up- or down-regulated in M versus interphase in a significant manner [142,147,148,150]. Because no one to date has monitored AURKA translation tightly over the cell cycle, we can only infer from studies that have used genome-wide approaches to compare the translatome of different cell cycle phases.

Qin & Sarnow [142] show that, while most mRNAs are found in polysome fractions of lower molecular weight in mitotic extracts compared with extracts from unsynchronized cells, 49 mRNAs (3%) remained associated with either more or a similar number of ribosomes, suggesting that translation of these is up-regulated or constant in M, respectively. The fact that AURKA cannot be found in this group of mRNAs might indicate that its translation decreases in M as for most cellular mRNAs. In support of this, Aviner *et al*. [145] found AURKA within the subset of 339 proteins (7%) that show statistically significant changes in translation rate between cell cycle stages, with a peak at G_2_/M. Integration of cell cycle transcriptome, translatome and total proteome data from HeLa cells indicated that, concordantly with the trend of AURKA mRNA abundance, AURKA translation rate also increases through S to peak at M and is minimal in G_1_ [146]. Conversely however, the absence of AURKA within a list of 1255 mRNAs (12%) that exhibit significantly different levels of ribosome occupancy in any cell cycle phase compared to other phases [148] would suggest that its translation runs at a pace that is similar throughout the cell cycle. A similar result revealed no alteration of AURKA translation efficiency in M compared to S phase [121]. It is worth noting that the last two reports are based on quantifications of ribosomal footprints on mRNAs (ribosome profiling): there is evidence that ribosome occupancy is not necessarily correlated with the rate of translation, as mechanisms might interfere with elongation speed without altering the abundance of residing polysomes [144,151–154]. Moreover, since ribosome profiling typically requires that the amount of ribosome-protected mRNA fragments be normalized to the abundance of the mRNA itself [155], one possible interpretation of the two studies is that the results only reflect changes in the rate of *AURKA* transcription. By contrast, the studies hinting that AURKA translation rate might change over the cell cycle are primarily based on quantification of the nascent protein, which could perhaps report on translational status in a more accurate manner in some contexts. Overall, while we do not know precisely when AURKA translation is activated in interphase, we believe it is not up-regulated but either constant or down-regulated in M phase compared with S and/or G_2_.

The general decrease in translation observed in M phase typically applies to cap-dependent translation, which is thought to be the preferred mechanism of protein synthesis, while Internal Ribosome Entry Site (IRES)-dependent translation is proposed to take over in this phase [156]. Assays with bicistronic RNA constructs revealed that AURKA 5′UTR contains an IRES element, whose activity is regulated in a cell cycle-dependent manner and peaks at G_2_/M phase in immortalized and cancer cells only, where AURKA cap-dependent translation remained unchanged [16]. Because there is no evidence so far that AURKA translation is enhanced in M phase, when IRES-mediated translation is thought to be most active, IRES-dependent translational activation of AURKA could be decoupled from generic IRES-dependent mitotic translation and could exclusively be relevant as a mechanism for AURKA overexpression in cancer [134]. What sequence or structural element precisely constitutes the AURKA IRES, and whether it has a physiological role in regulating AURKA expression, are still open questions.

### 5.2. Molecular mechanisms of *AURKA* translation

Molecular mechanisms controlling AURKA translation (figure 2*b*) have been uncovered in studies that only focus on pathological contexts, consequently giving insights into how translation contributes to expression of oncogenic AURKA.

In a search for mechanisms underlying enhanced AURKA protein expression in breast cancer, one report found that none of the processes of transcription, mRNA stability, cap-dependent translation and protein stability were responsible for overexpression in some immortalized and tumorigenic breast cell lines [16]. In these, activity of AURKA IRES element was found to be positively correlated with its protein levels, suggesting that a switch from cap- to IRES-dependent translation contributes to overexpression of AURKA at the level of translation, probably marking an early event during cancer progression. It is however not known what might cause the switch.

Another study reported that heterogeneous nuclear Ribo-Nucleoprotein Particle Q1 (hnRNP Q1), which is overexpressed in colorectal cancer and can promote cell proliferation, translationally up-regulates AURKA both in cap- and IRES-dependent manner via binding to AURKA 5′UTR [17]. hnRNP Q1 may also regulate AURKA protein expression in a cell cycle-dependent manner, since silencing of hnRNP Q1 decreased mitosis-dependent AURKA expression, and hnRNP Q1 overexpression increased AURKA abundance at G_2_/M phase. However, as this was only assessed by western blot, and since AURKA translation in M phase is likely not sustained, it is not excluded that hnRNP Q1 increases AURKA protein levels in M phase via a different mechanism than translational regulation. Additionally, the study confirmed that the activity of AURKA mRNA IRES was elevated in G_2_/M phase compared to G_1_/S phase in a cancer cell line (see above) and showed that the AURKA mRNA 5′UTR variants containing exon II bore stronger IRES activity than the variants containing exon I only. Because overexpression of hnRNP Q1 positively correlated with AURKA overexpression in human colorectal cancer tissues, the authors suggest that hnRNP Q1 may contribute to the tumorigenesis of colorectal cancer via AURKA translational up-regulation.

An earlier report from the same group had shown that translation of AURKA mRNA is up-regulated downstream of EGF signalling in EGFR-overexpressed colorectal cancer, as pulse-chase assays confirmed increased *de novo* AURKA protein synthesis and AURKA mRNA was found more associated with the ribosomal S6 protein upon EGF treatment [11]. The study also demonstrated that the PI3 K/Akt/mTOR and MEK/ERK pathways mediated the EGF-induced translational up-regulation of AURKA, and that 5′UTR splice variants containing exon II were critical for such up-regulation (see previous section). The pathway of translational upregulation downstream of EGF/EGFR signalling seems to exist in addition to the nuclear EGF/EGFR pathway that upregulates *AURKA* transcription [32]. Interestingly, a follow-up study found that hnRNP Q1 may be the factor that links EGF/EGFR signalling to AURKA translation [157], since treatment with EGF enhanced binding of hnRNP Q1 to AURKA mRNA, as well as the activity of hnRNP Q1 in.inducing AURKA translation. In addition, the mTOR and ERK pathways mediated hnRNP Q1-induced translation of AURKA mRNA upon EGF treatment.

Altogether, the regulation of AURKA translation may be much more complex than initially thought. As discussed so far, AURKA UTRs bear different elements that control expression at the level of translation, the role of many of such sequence and structural motifs, including miRNA binding sites, RNA-Binding Proteins (RBPs) recognition sites and RNA modifications in the regulation of AURKA translation are unexplored to this date. Fortunately, several *in vivo* methods are being developed recently, such as Translating RNA Imaging by Coat Protein Knock-off (TRICK) [158] or Nascent Chain Tracking (NCT) techniques [159], that track translation with high temporal and spatial precision and allow to probe the functional interaction between mRNA sequence elements and potential regulators.

## 6. Integrated temporal view of *AURKA* expression

**Figure 3.**
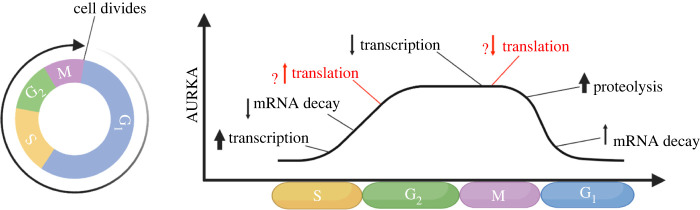
Different stages of gene expression integrate into AURKA temporal expression. Activation of AURKA transcription and protein degradation are likely the drivers of the respective increase and decrease in AURKA protein levels during the cell cycle. Control of mRNA stabilization also contributes to AURKA expression pattern, whereas the precise timing and extent of AURKA translational activation and translational inhibition are not yet clear. Figure created using BioRender.com.

The regulatory steps of transcription, post-transcription, translation and post-translation combine to confer *AURKA* gene its characteristic cell cycle-dependent pattern of expression (figure 3). In the late 1990 s, initial research addressed the trend of AURKA protein expression during the cell cycle in mammalian and human cells, although only qualitatively [19,23,93]. In parallel, first studies on AURKA protein degradation elucidated that, at the end of M phase, AURKA protein is maximally degraded by the APC/C linked to the Ubiquitin Proteasome System (UPS) [160]. The pattern of *AURKA* expression during the cell cycle was later confirmed quantitatively [161], and DNA microarray analyses consistently found *AURKA* in the G_2_/M cluster of transcriptionally co-regulated genes [56,67,162]. Most recent analysis of time-resolved profiling of the cell cycle transcriptome using the FUCCI system confirmed *AURKA* to be downregulated during M/G_1_ transition [57]. *AURKA* is also among the genes with the highest cell cycle periodicity [56,66,94]. However, how transcription combines with mRNA stability, translation and protein dynamics to control AURKA's pattern of expression is not fully understood, especially in terms of the extent and the timing of the individual contributory mechanisms.

There is consolidated evidence that AURKA protein and mRNA levels are extremely low in G_1_ phase and start accumulating in S phase, to then peak at G_2_/M (figure 3). Indeed, S is the phase where AURKA transcription is switched on first by de-repression and then by activation (see above). For this, transcription is a key contributor to increasing AURKA levels during S until M. Mechanisms of mRNA stabilization might intervene in this phase to also facilitate the increase in mRNA abundance. Accordingly, Battich *et al.* [163] categorized *AURKA* amongst genes whose mRNA abundance results from a cooperative strategy between the rate of synthesis and that of degradation of mRNA, that is, increase in synthesis rate accompanied by a decrease in degradation rate, and vice versa. As soon as AURKA mRNA is transcribed and fully processed, translation initiates. Because the *de novo* transcribed AURKA mRNA copies can already appear in G_1_ [161], this could be the earliest time in which translation can begin. Although it has not been entirely proven, at this time translation of AURKA mRNA is probably only basal, to be actively enhanced later in S phase [146]. Once translation initiates, the contribution of the rate of protein stabilization must be considered as well, although the regulation of AURKA proteolysis in interphase is less understood compared with its APC/C^FZR1^-mediated degradation in M phase [164]. However, because the very peak of AURKA expression is reached only in late G_2_ (i.e. not immediately after the activation of transcription), eventual mechanisms of mRNA stabilization, translational enhancement and protein stabilization may altogether contribute less than transcription to rising AURKA levels in S and G_2_.

During M phase the whole program of AURKA expression changes abruptly to a shutdown, as the aim for the dividing cell now is to irreversibly eliminate mitotic AURKA activity in a very short time. This in fact represents a tightly regulated step during exit from M phase. Ubiquitin-mediated proteolysis is important for the decline in AURKA protein levels during this time [164]. AURKA degradation in M phase is strictly dependent upon the FZR1-activated version of APC/C [165,166]. Furthermore, *AURKA* transcription is turned off during M phase. While transcriptional arrest eventually contributes to a drop in mRNA levels, turnover of the existing pool of AURKA mRNAs must be accelerated. It is reasonable to assume that AURKA mRNA undergoes canonical mRNA decay pathways, however very little is known about what controls AURKA mRNA stability. By investigating the hypothesis that mRNA decay might be important to reset cell cycle gene expression at mitotic exit, similarly to timed protein degradation, Krenning *et al.* [57] identified two temporal ‘waves’ of mRNA decline during M-G_1_: one of immediate decrease that initiates during anaphase and one of delayed decrease set off during early G_1_. AURKA mRNA was found in the delayed decrease group, as its levels start to decline 1–4 h after the start of G_1_ with a computed half-life of about 40 min in this phase. The fact that the half-life of AURKA mRNA during this cell cycle window is shorter than that measured in asynchronously growing cells [44,57,63] suggests programmed mRNA degradation in early G_1_.

In sum, just as activation of transcription is the driver for the increasing AURKA levels in early S, activation of protein degradation is key to disappearance of AURKA protein in M. However, S phase transcription is assisted by mechanisms favouring mRNA stabilization, translation and protein stabilization, which become more prominent as the cell progresses through G_2_, whereas mitosis-dependent proteolysis is accompanied by the shutdown of transcription and of translation and enhancement of mRNA decay. These latter events seem to remain in place in G_1_ even after AURKA proteolysis is concluded, so that the absence or low levels of AURKA are ensured in early interphase of the daughter cells. Except for transcription and proteolysis, the molecular mechanisms responsible for the activation/repression switch of translation and mRNA decay have to date never been explored.

## 7. Conclusion

This literature review highlights how AURKA expression is tightly regulated at multiple levels to adopt a pattern strictly correlated to the cell cycle. Needless to say, understanding the mechanisms regulating AURKA gene expression is an important quest in the study of the eukaryotic cell cycle itself. At the transcriptional level, AURKA is controlled by several molecular mechanisms and transcription factor complexes, which ensure its timely activation in S phase and repression in M phase. *AURKA* promoter also responds to external stimuli such as oxygen levels and presence of growth factors. Post-transcriptionally, splicing of the 5′UTR accounts for the existence of at least 16 different mRNA isoforms, and alternative cleavage and polyadenylation generates two different 3′UTR isoforms. Only now are we starting to gain an understanding of how these isoforms control both AURKA mRNA and protein dynamics, and how they are involved in disease. Translation of AURKA remains a less explored step of gene expression, but recent analyses of cell cycle translatomes raise our expectations of the existence of active translational regulatory mechanisms for AURKA mRNA. Not to mention the increasingly recognized role of translational dysregulation in disease, some examples of which have been already reported for AURKA. Even though we could qualitatively assess the contribution of DNA, mRNA, and protein dynamics to the cell cycle-dependent expression of AURKA, several questions remain to be addressed. On the one hand, the cell cycle pattern of AURKA expression follows that of many other cell cycle genes, especially those with temporally overlapping cell cycle functions. On the other hand, some mechanisms of post-transcriptional control, for example, AS and alternative polyadenylation, might be exclusive to AURKA mRNA. Post-transcriptional regulatory events may specifically occur in interphase or in non-cycling cells, where AURKA exerts functions not related to cell division. It is plausible to state that the definition of AURKA as a ‘G_2_/M’ gene only refers to its pattern of gene expression in dividing cells, not to its period of activity. It is now broadly accepted that AURKA plays other physiological cell- and tissue-specific roles that are independent of mitosis (e.g. occur in G_1_ or S), of protein abundance (e.g. occur when AURKA expression is low) and of cellular proliferation rate (e.g. occur in non-dividing cells). Moreover, it is possible that there exist some non-pathological dividing cell populations in which AURKA is not classifiable as ‘G_2_/M’. Addressing the open questions highlighted in this review will be crucial to discern which stage of gene expression would be more efficient to target when the aim is to correct AURKA abundance in cancers where it is altered. Quantitatively and qualitatively aberrant AURKA expression in patients with different cancers have been observed at all levels of gene expression. This can only motivate deeper investigations into AURKA expression.

## Data Availability

This article has no additional data.
